# Detection of morphologic alterations in rectal carcinoma following preoperative radiochemotherapy based on multiphoton microscopy imaging

**DOI:** 10.1186/s12885-015-1157-5

**Published:** 2015-03-18

**Authors:** Lianhuang Li, Zhifen Chen, Xingfu Wang, Hongsheng Li, Weizhong Jiang, Shuangmu Zhuo, Guoxian Guan, Jianxin Chen

**Affiliations:** 1 Institute of Laser and Optoelectronics Technology, Fujian Provincial Key Laboratory for Photonics Technology, Key Laboratory of OptoElectronic Science and Technology for Medicine of Ministry of Education, Fujian Normal University, Fuzhou, 350007 China; 2 Department of Colorectal Surgery, The Affiliated Union Hospital, Fujian Medical University, Fuzhou, 350001 China; 3 Department of Pathology, The First Affiliated Hospital, Fujian Medical University, Fuzhou, 350001 China

**Keywords:** Multiphoton microscopy, Preoperative radiochemotherapy, Fibrosis, Two-photon excited fluorescence, Second harmonic generation

## Abstract

**Background:**

Preoperative radiochemotherapy improves outcomes in patients with locally advanced rectal carcinoma, and has been used increasingly in patient management. However, there is a strong clinical need to assess tumor response to neoadjuvant treatment, and a non-invasive technique that allows the precise identification of morphologic changes in tumors would be of considerable clinical interest.

**Methods:**

In this study, we used multiphoton microscopy (MPM) to detect morphologic alterations in rectal adenocarcinomas in patients treated with preoperative radiochemotherapy.

**Results:**

MPM was able to identify histopathologic alterations in rectal cancer following preoperative radiochemotherapy, and allowed the qualitative assessment of treatment efficacy and feasibility in relation to dose or strategy.

**Conclusion:**

These findings may provide the groundwork for evaluating tumor response to neoadjuvant treatment, thus allowing the tailoring of effective treatment doses and strategies.

## Background

Accumulating evidence has demonstrated that neoadjuvant treatment improves local control and survival in patients with rectal cancer, and may play an increasing role, especially in locally advanced disease [1-5]. Preoperative radiochemotherapy has thus been used increasingly in the management of this group of patients. Pathological assessment based on the histopathological investigation of resected specimens is important for estimating the prognosis and effect of radiochemotherapy [6,7]. However, pathological examination is associated with several disadvantages, such as crush artifacts, bleeding, sampling errors, and time-consuming pathological procedures [8,9]. In contrast, multiphoton microscopy (MPM), which is based on intrinsic two-photon excited fluorescence (TPEF) and second harmonic generation (SHG), offers significant advantages for imaging in thick tissue and live animals, including greater imaging penetration depth, reduced out-of-focus photobleaching and phototoxicity, and the ability to detect the cellular and subcellular microstructures of biological tissues [10-12].

This study therefore investigated the treatment-related morphologic aspects of rectal carcinomas using MPM imaging, with an emphasis on stromal alterations, changes in blood vessels and inflammatory cell infiltrate, and residual tumor cells. The study aimed to provide a detailed morphologic description of rectal carcinoma in patients treated with preoperative radiochemotherapy, and to identify patterns of morphologic alterations with prognostic significance. We also aimed to determine the efficacy of radiochemotherapy and the appropriateness of the treatment dose and strategy by monitoring theses morphological changes.

## Methods

### MPM imaging system

The MPM imaging system used in this study has previously been described in detail [13,14]. Briefly, MPM images were acquired using a LSM 510 META system (Zeiss, Jena, Germany) coupled to a Ti:sapphire laser (Mira 900-F, Coherent Inc., Santa Clara, CA, USA). An oil immersion objective (Plan-Apochromat 63×, N.A.1.4) was used to focus the excitation beam into samples and to collect the back-scattered TPEF/SHG signals. Two different channels were selected to obtain high-contrast images of collagen and fluorescence components, respectively. One channel corresponded to wavelengths of 387–419 nm to reveal the collagen microstructure using SHG signals, while the other channel covered wavelengths 430–716 nm to reveal the morphology of fluorescence components using TPEF signals at an excitation wavelength of 810 nm. The contrast of the SHG/TPEF images was increased by color-coding the SHG images green and the TPEF images red.

### Specimen preparation

This investigation was approved by the Institutional Review Board of the Affiliated Union Hospital, Fujian Medical University and conformed with the institutional rules governing clinical investigations of human subjects in biomedical research. Prior to study participation, all patients signed an informed consent form. All patients underwent long-term preoperative radiochemotherapy (45 Gy/25 fractions followed by a 5.4 Gy boost, for a total of 50.4 Gy, and oral capecitabine 825 mg/m^2^ twice daily during radiotherapy). According to the Chinese guidelines for colorectal cancer treatment, radical surgery was performed about 8 weeks after the end of radiotherapy. Patients with different responses to therapy were selected, and patients who failed to respond were excluded. Tumor response to therapy was judged macroscopically in harvested specimens.

Seven fresh tumor samples were obtained from seven patients undergoing rectum resection after preoperative radiochemotherapy at the Affiliated Union Hospital of Fujian Medical University. Normal specimens were collected 6 cm outside the cancer margin. Patient ages ranged from 38–67 years (53 ± 10 years) and the male/female ratio was 2.5. Detailed information, including cancer classification and clinical stage, was shown in Table 1. Five serial tissue slices (10 μm thick) were cut from each specimen and the middle slice was processed for histological examination with hematoxylin and eosin (H&E) stain, according to standard histology procedures. The other tissue sections were sandwiched between a microscope slide and glass coverslip for MPM imaging. The tissue slices were imaged with the coverslip facing the microscope objective. Phosphate-buffered saline solution was dripped into the specimen during imaging to avoid dehydration or shrinkage during the imaging process.

**Table 1:** **Patient information including cancer classification and clinical stage**

Patients	Cancer classification	Clinical stage
1	Adenocarcinoma	cT2N + M0, Stage III
2	Adenocarcinoma	cT3N + M0, Stage III
3	Adenocarcinoma	cT3N + M0, Stage III
4	Adenocarcinoma	cT3N0M0, Stage II
5	Adenocarcinoma	cT3N + M0, Stage III
6	Adenocarcinoma	cT3N + M0, Stage III
7	Adenocarcinoma	cT3N + M0, Stage III

### Histology

H&E-stained sections were reviewed by a certified pathologist. Images were obtained by standard bright-field light microscopy (Eclipse Ci-L, Nikon Instruments Inc., Japan) with a charge-coupled device (Nikon, DS-Fi2). Finally, the MPM and corresponding H&E-stained images (40×) were analyzed and compared by a certified pathologist.

### Quantification of morphological features

Quantitative changes in fibrotic tissue, cellular architecture, and blood vessels during rectal carcinoma progression following preoperative radiochemotherapy were assessed by calculating the collagen density, nuclear area, and vessel wall thickness, respectively. For each blood vessel, three random positions were selected and the vessel wall thickness was determined as the mean thicknesses of the three positions. Collagen density was defined as the ratio of SHG to all pixels in each image. The nuclear area was calculated by measuring the area enclosed by the nuclear boundary. Values were expressed as means and standard deviations (mean ± SD). The standard deviation signified the change in nucleus size or vessel wall thicknesses, respectively.

## Results

### Stromal alteration

**Figure 1:**
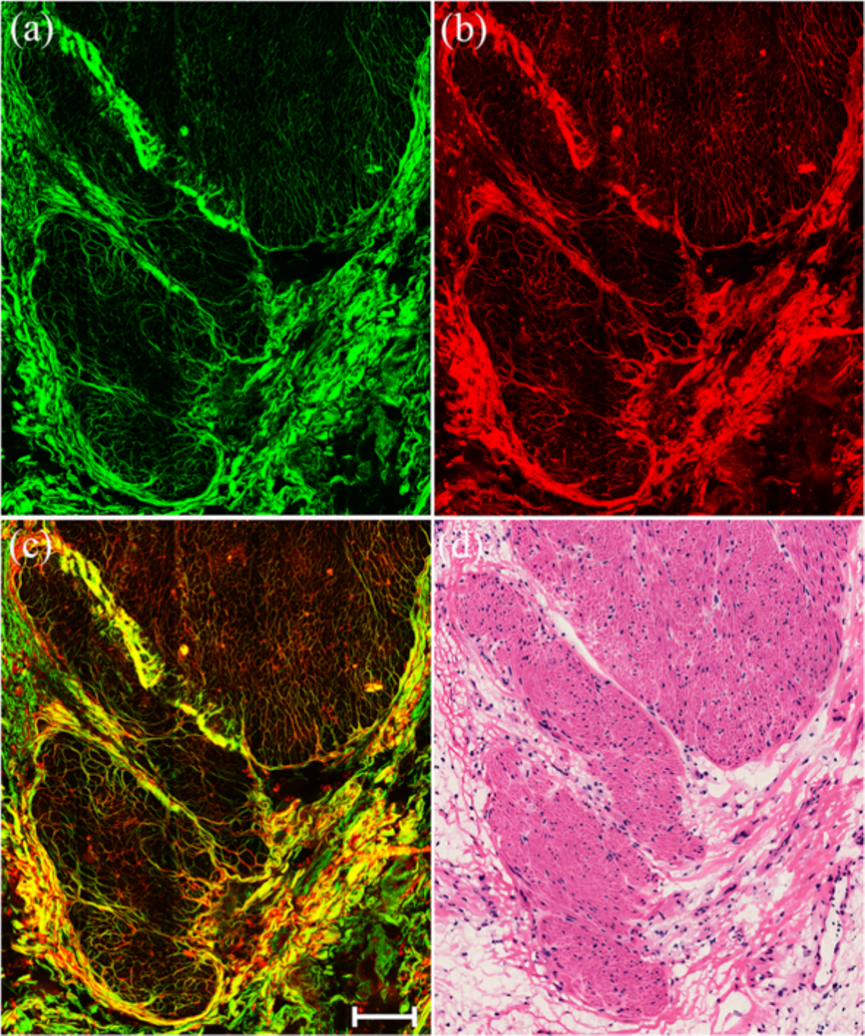
**Representative TPEF/SHG images of stromal alterations in rectal carcinoma after preoperative radiochemotherapy characterized by fibrosis or fibroinflammatory changes.** Scale bar = 100 μm. **(a)** SHG image (green); **(b)** TPEF image (red); **(c)** overlay of SHG/TPEF images; and **(d)** corresponding H&E-stained image (40× magnification).

Representative MPM and corresponding H&E-stained images showed stromal alterations in rectal carcinomas induced by preoperative radiochemotherapy with cancerous cell invasion into the muscularis propria (Figure 1). MPM images clearly revealed stromal changes in rectal cancer patients undergoing preoperative radiochemotherapy. Consecutive muscular tissues were disrupted and collagen fibers were abundant but disordered, as demonstrated by SHG signals (green) (Figure 1(a)). This may be interpreted as former tumor infiltration leading to the destruction of muscular tissues, and post-treatment tumor regression represented by fibrosis or fibroinflammatory changes replacing neoplastic glands [15,16]. These fibrotic tissues also produced comparable TPEF signals (red) (Figure 1(b)), and overlaid TPEF/SHG images therefore appear yellowish (Figure 1(c)). The details revealed by MPM correlated well with the H&E-stained images (Figure 1(d)).

### Changes in blood vessels and inflammatory cell infiltrate

**Figure 2:**
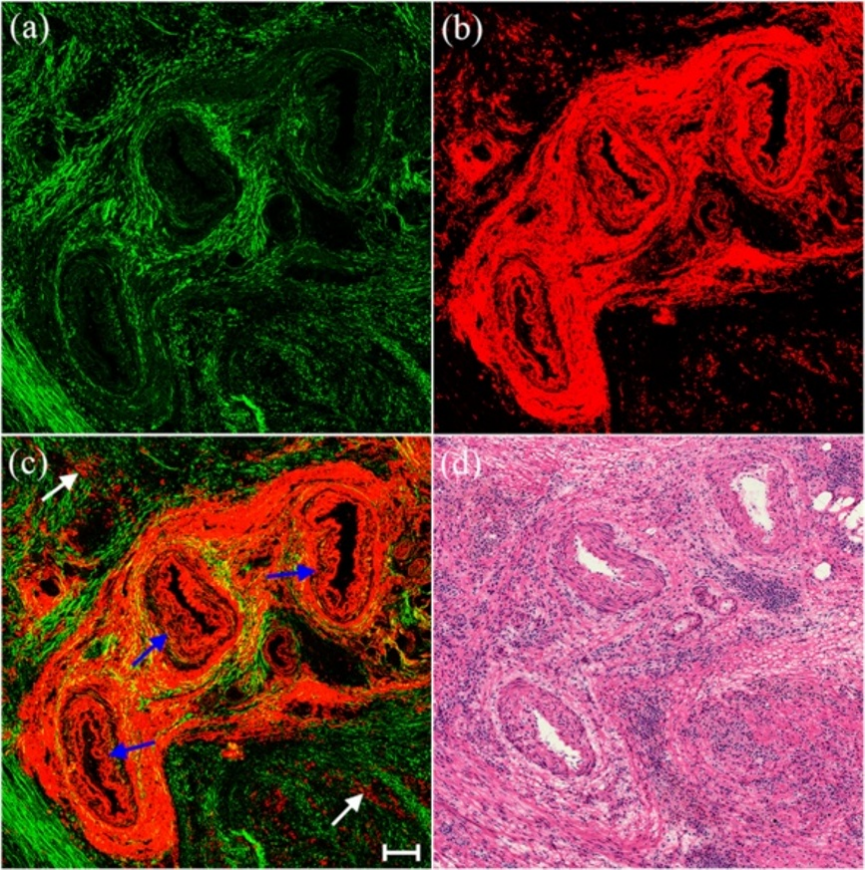
**Representative TPEF/SHG images of blood vessel changes with thickening and fibrosis of the intima and media, and inflammatory cell infiltration.** Scale bar = 100 μm. **(a)** SHG image (green); **(b)** TPEF image (red); **(c)** overlay of SHG/TPEF images; and **(d)** corresponding H&E-stained image (40× magnification).

MPM and H&E staining revealed obvious radiogenic blood vessel changes and inflammatory cell infiltration in the serosa after preoperative radiochemotherapy (Figure 2). The blood vessels showed significant alterations with thickening and fibrosis of the intima and media, as shown by TPEF signals in MPM (Figure 2(c)) (blue arrows) [7]. TPEF signals also revealed inflammatory cell infiltration and large numbers of inflammatory cells infiltrating into the stroma (Figure 2(c)) (white arrows). The tumor-associated inflammatory reaction has long been considered as a type of host response and an important factor in tumor progression [16]. Previous studies also demonstrated lower recurrence rates and better outcomes in patients with rectal cancer who had abundant inflammatory cells in the stroma post-irradiation [17,18]. These qualitative morphological variations were consistent with the paired histological sections in the current study (Figure 2(d)).

### Residual tumor cells

**Figure 3:**
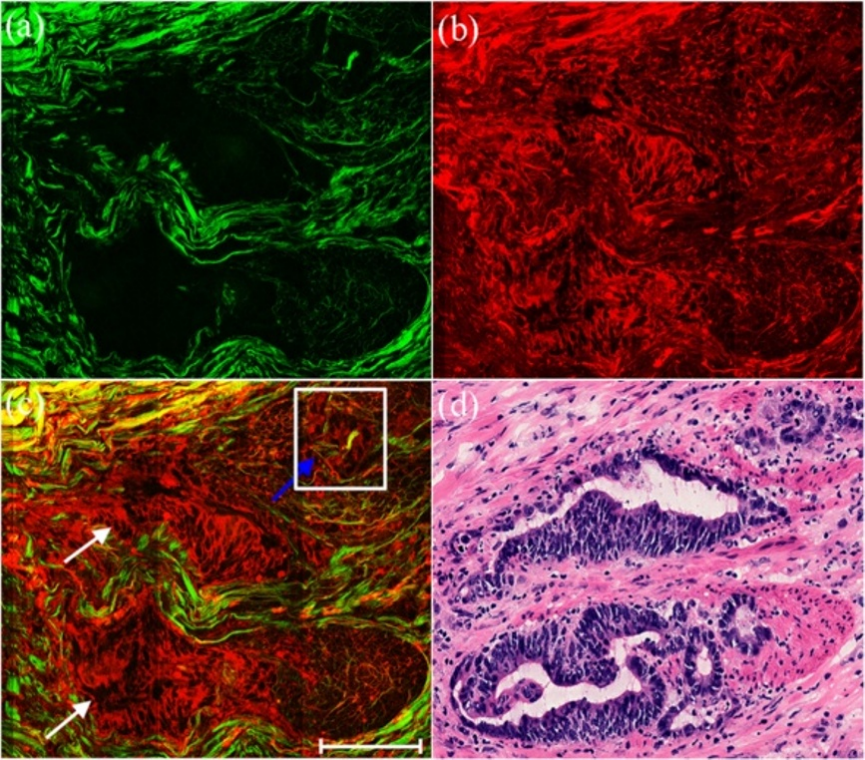
**Representative TPEF/SHG images of residual tumors surrounded by fibrosis with minimal inflammatory cells.** Scale bar = 100 μm. **(a)** SHG image (green); **(b)** TPEF image (red); **(c)** overlay of SHG/TPEF images; and **(d)** corresponding H&E-stained image (40× magnification).

**Figure 4:**
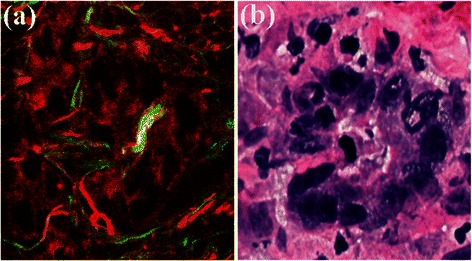
**Magnification of region of interest in Figure****3****(c) (white box) and corresponding H&E-stained image. (a)** MPM image; and **(b)** H&E-stained image.

MPM and H&E-stained images showed residual tumors in the muscularis propria after preoperative radiochemotherapy (Figure 3). Tumor cells in the rectal carcinoma may show marked posttreatment changes, such as nuclear atypia, and these altered tumor cells may retain a glandular growth pattern or become more solid [15,16]. In the current study, the dominant tumor morphologic pattern remained similar in treated and untreated rectal adenocarcinomas (white arrows); the tumors were surrounded by fibrosis with minimal inflammatory cells, while some tumor cells became solid (blue arrow). Furthermore, MPM allowed the differentiation of cellular features such as nuclear pleomorphism, which is an important biologic characteristic reflecting tumor grade, degree of differentiation, and proliferation. The region of interest within the white box in Figure 3(c) is magnified in Figure 4 to show the ultrastructure of the residual cancerous cells more clearly. These alterations following preoperative radiochemotherapy are common and might be clinically meaningful. The details of the morphological changes revealed by MPM correlated well with those shown in H&E-stained images (Figure 3(d)).

### Quantitative analysis of morphological features

Changes in the morphological features of rectal carcinomas treated by radiochemotherapy were described quantitatively by analyzing collagen density, vessel wall thickness, and nuclear area (Table 2).

**Table 2:** **Morphological features of rectal carcinoma after preoperative radiochemotherapy**

	Patient no.							
Morphologic features	1	2	3	4	5	6	7	Average
Collagen density of normal submucosa	0.78	0.81	0.85	0.75	0.79	0.67	0.71	0.77 ± 0.06
Collagen density of normal serosa	0.69	0.67	0.75	0.70	0.79	0.63	0.74	0.71 ± 0.05
Collagen density of fibrosis associate with carcinoma	0.97	0.96	1.00	0.92	0.99	0.95	0.98	0.97 ± 0.03
Nuclear area (μm^2^) of carcinoma	197.51	89.18	74.63	103.86	42.67	44.34	30.77	83.28 ± 57.02
Vessel wall thickness (μm) of carcinoma	71.50	89.15	130.69	162.96	150.50	135.65	80.13	117.23 ± 36.46

*P < 0.0001 (collagen density between the submucosa, serosa and fibrosis), and P > 0.05 (collagen density between the submucosa and serosa).

**Figure 5:**
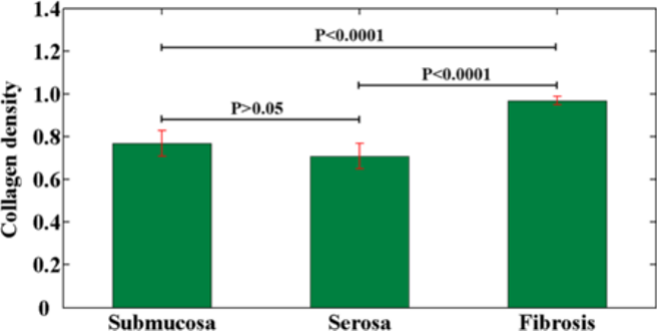
**Collagen density in stromal fibrosis, normal submucosa, and serosa.** Error bars indicate SD.

The collagen density in stromal fibrosis (0.97 ± 0.03) was significantly higher compared with normal submucosa (0.77 ± 0.06) and serosa (0.71 ± 0.05) (Figure 5) (P < 0.0001 between the submucosa, serosa, and fibrosis: one-way ANOVA test; SPSS 15.0). The vessel wall thickness was 117.23 ± 36.46 μm, and the relatively large SD indicates considerable variation in blood vessel size. The nuclear area was 83.28 ± 57.02 μm^2^, and again the large SD demonstrates that carcinomatous cells in post-treatment rectal carcinoma displayed marked nuclear atypia. A combination of collagen density, vessel wall thickness, and nuclear area may thus be useful for quantitatively monitoring the development of rectal carcinomas in patients undergoing preoperative radiochemotherapy.

## Discussion

Preoperative radiochemotherapy has been shown to be useful for reducing tumor size and increasing operability. More importantly, preoperative radiotherapy in combination with surgery has been shown to decrease the rate of local recurrence in rectal cancer patients [17-19]. Preoperative radiochemotherapy can modify the histologic appearance of rectal cancer in terms of fibrosis, colloid response (data not shown), blood vessel hyperplasia, and inflammatory reaction, and these morphologic alterations have shown clinically meaningful correlations with patient outcome [7,16]. Although these changes can be determined by histological examination of resection specimens, the procedure is troublesome and time-consuming.

Fortunately, fibrous tissues consist mainly of collagen fibers and emit strong SHG signals, whereas blood vessels, residual carcinomatous cells, and inflammatory cells contain abundant elastin and generate TPEF signals [20,21]. These signals make it possible to detect histopathologic changes using MPM. The current study employed MPM to provide a detailed morphologic description of rectal carcinomas in patients undergoing preoperative radiochemotherapy. The results demonstrated that MPM imaging was able to identify blood vessel hyperplasia and tumor regression by the disappearance of carcinoma cells and their replacement by fibrous or fibroinflammatory tissues. Furthermore, the high resolution of MPM to approximately the cellular/sub-cellular level [22,23], enables this technique to identify residual carcinomatous cells and distinguish between tumor cells and other cells with different morphologies, such as adipose cells [24].

There is currently no validated method for directly monitoring the efficacy of radiochemotherapy, and the appropriateness of the treatment dose and strategy. The current study showed that radiochemotherapy caused morphological changes in rectal carcinomas, including changes in nuclear shape, collagen density, and vessel wall thickness, and these changes could be identified and quantitatively described by MPM. These results suggest that MPM may be a useful tool for evaluating tumor response to neoadjuvant treatment by enabling monitoring of the morphological changes and subsequent tailoring of the effective treatment dose or strategy.

Radiochemotherapy is a standard approach in advanced solid human tumors. Tumors often develop in the mucosal layer and gradually infiltrate to the lamina propria, submucosal layer, and deeper layer of the bowel wall. The cancer is therefore most advanced in the superficial layer, and the curative effect of radiochemotherapy can be evaluated qualitatively by monitoring treatment-related morphological changes in the superficial layer. MPM has been reported to penetrate to depths of millimeters [25]. MPM may therefore be useful for determining if radiochemotherapy has a curative effect, and if the treatment dose and strategy are appropriate regardless of cancer stage. Subsequent treatments can then be tailored to meet the specific clinical needs of different patients.

There are some drawbacks associated with this technique, and some limitations of the current study. There are three major factors limiting the *in vivo* application of MPM. First, the limited field of view makes examination of large areas or volumes problematic, and may lead to interobserver variability. Second, the depth of imaging is limited, though this limitation may be overcome by the continuous advancement of a gradient index lens-based MPM. Finally, the technique is expensive, though it is possible that the cost may be reduced by using a combination of fiber laser and MPM. The study limitations included the small patient number and lack of comparison of pre- and post-therapeutic samples. However, these factors do not affect the conclusion that MPM can be used to monitor tumor response to preoperative radiochemotherapy.

The importance of the impact of histopathological factors on prognosis and an awareness of the role of morphologic alterations in ensuring an accurate pathologic assessment [16,26,27] indicate the urgent need for a new, label-free, real-time, noninvasive technique for differentiating among the various morphologic alterations induced by radiochemotherapy. The results of the present study suggest that MPM might provide a real-time, label-free technique for evaluating tumor response following preoperative radiochemotherapy and for noninvasive, *in vivo* pathophysiological analysis. The advantages of MPM indicate that it may be useful for the *in vivo* assessment of treatment effect, dose, and strategy in patients with rectal carcinoma receiving preoperative radiochemotherapy.

## Conclusions

MPM can be used to differentiate morphologic changes in rectal carcinomas in patients undergoing preoperative radiochemotherapy. The advancement of clinically miniaturized MPM and multiphoton probes to allow MPM to be combined with standard endoscopes will allow the real-time *in vivo* evaluation of tumor response to neoadjuvant therapy and the subsequent tailoring of effective treatment doses and strategies.
